# Morphology and Microstructure of As-Synthesized Anodic TiO_2_ Nanotube Arrays

**DOI:** 10.1007/s11671-010-9812-x

**Published:** 2010-10-07

**Authors:** Chunbin Cao, Guoshun Zhang, Xueping Song, Zhaoqi Sun

**Affiliations:** 1 School of Sciences, Anhui Agricultural University, Hefei, 230036, China; 2 School of Physics and Material Science, Anhui University, No. 3, Feixi Road, Hefei City, Anhui Province, 230039, China

**Keywords:** Anodization, TiO_2_ nanotube arrays, Microstructure, Growth mechanism

## Abstract

The as-grown structure of electrochemically synthesized titania nanotube arrays is investigated by scanning electron microscope (SEM) in combination with transmission electron microscope (TEM) as well as X-ray diffraction (XRD). The analysis reveals a preferred growth direction of the nanotubes relative to the substrate surface and the well control on the nanotube arrays morphology. The crystal structure of the anatase phase is detected and exists in the tube walls without any thermal treatment, which makes it possible to realize the application of as-formed TiO_2_ nanotubes avoiding the degradation of the nanotube structures when sintering. In addition, a new growth, layered model of the anodic TiO_2_ nanotubes is presented to obtain further understanding of the growth mechanism.

## Introduction

Highly ordered and high surface area TiO_2_ nanotube arrays have attracted much attention for their potential use in several practical applications, such as photocatalytic applications [1,2], sensing [3,4], photoelectrolysis [5,6], polymer-based bulk heterojunction photovoltaics [7,8], dye-sensitized solar cells [9,10], biofluids filtration, drug delivery and other biomedical applications [11,12]. It is known that the as-fabricated nanotube arrays commonly have an amorphous crystallographic structure. After annealing at elevated temperatures in atmosphere, the nanotube walls transform into anatase phase, and a layer underneath the nanotubes converts into rutile [13-17].

TiO_2_ properties depend on the crystallinity and hence the utility of their applications also varies. For example, anatase phase is preferred in charge-separating devices such as dye-sensitized solar cells and in photocatalysis, while rutile is used predominantly as dielectric layers and in gas sensors. However, due to nucleation growth type of phase transformations, porosity and/or surface area reduction occur with the sintering [18]. Yang and co-workers made the observations that the nanotube length decreased when calcination temperature is higher than 550°C and completely collapsed at 800°C [19]. So, it is significant to fabricate TiO_2_ nanotube arrays with applicable crystallinity and isomorph type structures at low temperature. In this work, we report on the crystallinity in the nanotube layer after anodization without any thermal treatment. By analysis on current curve in conjunction with the SEM and TEM images, a layered growth model is put forth to obtain further understanding of anodic TiO_2_ nanotube arrays formation.

## Experimental

The Ti foils (0.1 mm, 99.6% purity) were degreased prior to anodization by sonicating in acetone, rinsed with deionized water (DI). The electrochemical set-up consisted of a high-voltage potentiostat Jaissle IMP 88 and a classical two-electrode cell, leaving the electrodes distance 1 cm and Ti surface 1 cm^2^ open to the electrolyte. The samples 1, 2 and 3 were anodized in solutions containing 0.175 M NH_4_F consisting of mixtures of DI water and glycerol (volume ratio 3:97%) at 30 V for 3, 6 and 12 h, respectively. The sample 4 was prepared in mixed electrolyte containing DI water and glycerol (volume ratio 50:50%) and 0.175 M NH_4_F at 20 V for 2 h. The sample 5 was anodized in glycerol: 0.175 M NH_4_F at 30 V for 3 h. A scanning electron microscope Hitachi FE-SEM S4800 and a transmission electron microscope (TEM) JEM-2100 were employed for the morphological characterization of the TiO_2_ nanotubular layers. The crystalline structures of the TNT arrays were checked by means of X-ray diffraction (XRD, MAC M18XHF).

## Results and Discussion

### Morphology

**Figure 1:**
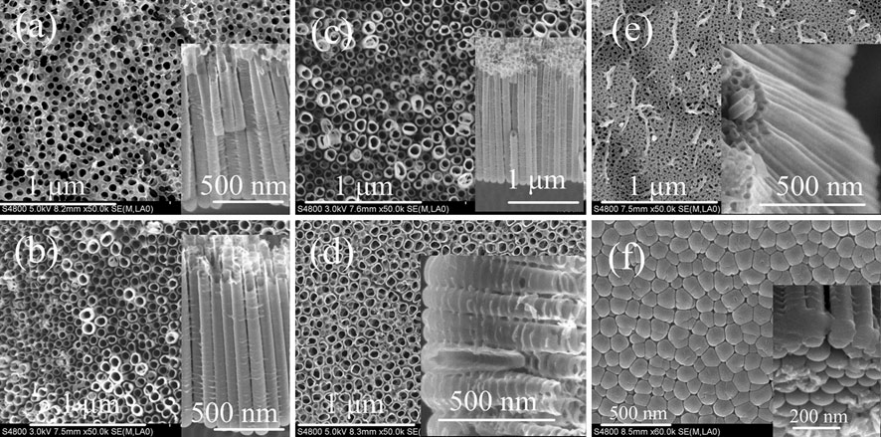
***a–e* SEM *top views* (and *cross-sections* in insets) of samples 1, 2, 3, 4 and 5, respectively; *f* SEM *bottom view* (and *bottom side view* in inset) of sample 1**.

The anodic conditions and morphology parameters of the nanotubes, prepared in the electrolytes containing 0.175 M NH_4_F and different volume ratios of water and glycerol, are listed in Table 1. SEM top view of sample 1, in Figure 1a, shows the porous film-like surface. The pits, with the obvious distance, indicate that the initial oxide layer has not been completely dissolved, but underneath which the TiO_2_ nanotubes have already been formed. Similar phenomenon can also be seen in sample 5, in Figure 1e, and in the inset, neither gaps among the tubes nor are ridges on the side wall found. Sample 4, fabricated in the electrolyte containing more water, in Figure 1d, exhibits the thinner tube wall but the smoother layer surface (contrasted with those of samples 2 and 3).

**Table 1:** The anodic conditions and morphology parameters of the nanotubes prepared in the electrolytes containing 0.175 M NH_4_F and different volume ratios of water and glycerol

Samples	Electrolytes	Voltage (V)	Anodic time (h)	Tube length (nm)	Tube mouth diameter (nm)
1	Water/glycerol (Vol. 3:97%)	30	3	1,190 ± 10	–
2			6	1,240 ± 20	60 ± 5
3			12	1,720 ± 20	70 ± 5
4	Water/glycerol (Vol. 50:50%)	20	2	570 ± 10	80 ± 5
5	Water/glycerol (Vol. 0:100%)	30	3	830 ± 10	–

Figure 1f shows the SEM bottom and bottom side (inset) views of sample 1. The tube bottom exhibits the hexagon-like cell morphology. Que Anh S. Nguyen and co-workers reported that the separation of the nanotubes from the substrate most likely occurs along the barrier layer/nanotube interface rather than along the titanium substrate/nanotube interface [20]. In this work, by mechanically bending the samples, the separated nanotube arrays were obtained, and the clear bottoms are shown in Figure 1f (inset). But from the bared Ti substrate, no oxide layer has been found.

### Microstructure

**Figure 2:**
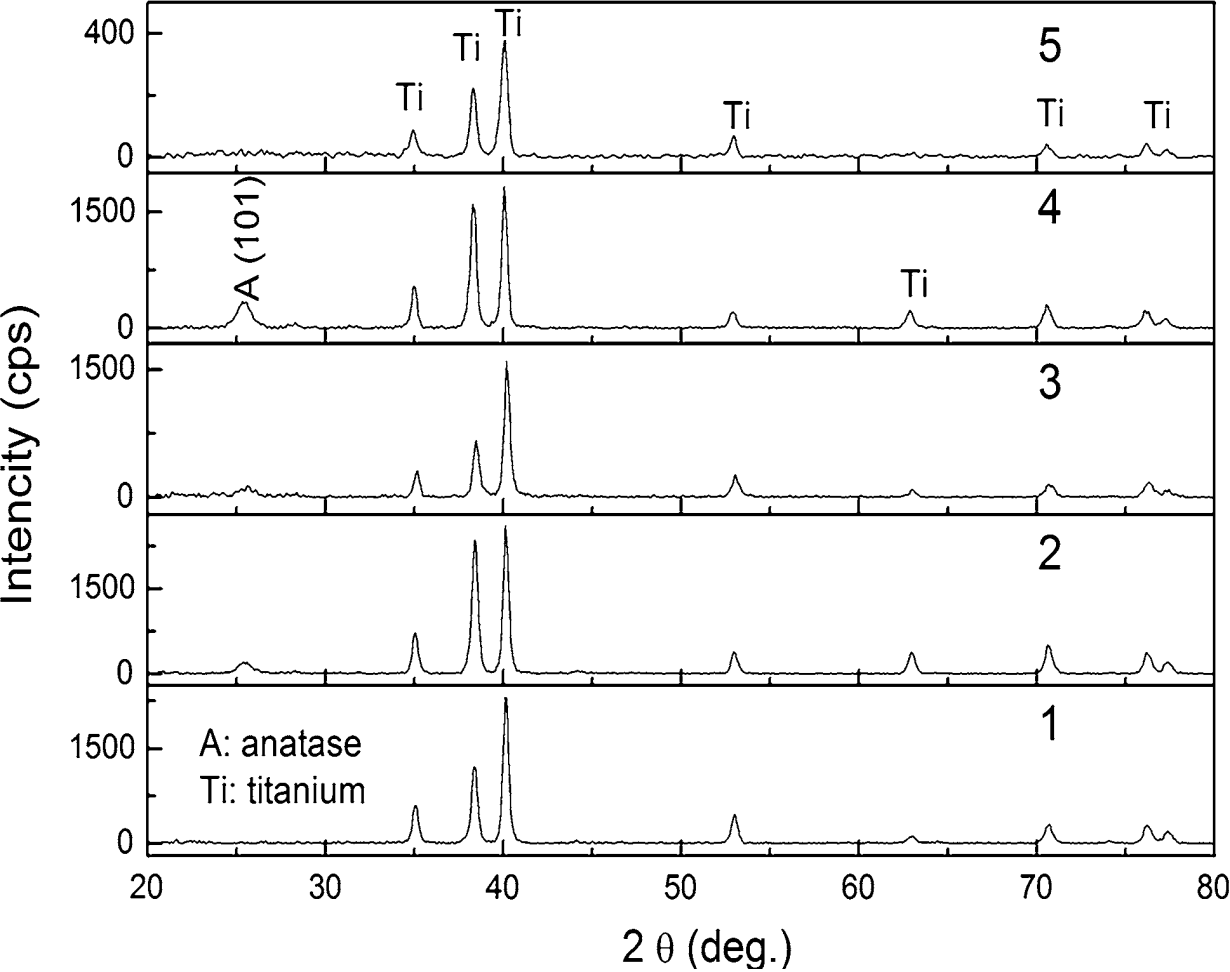
**XRD patterns of all samples (X-ray glancing angle 4°)**.

**Figure 3:**
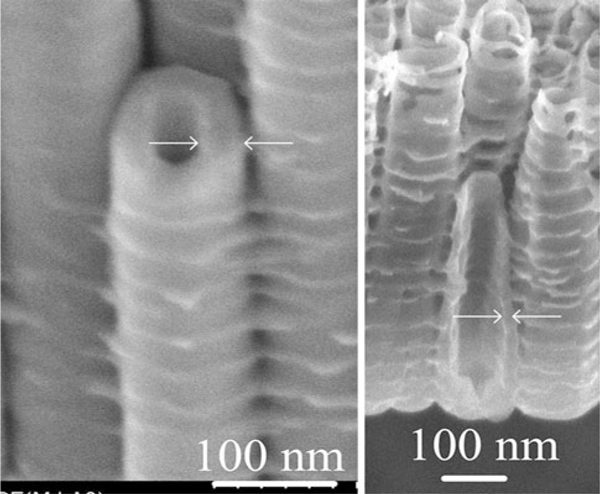
**SEM images showing tube wall thicknesses of sample 3 (*left side*) and sample 4 (*right side*)**.

As-formed TiO_2_ nanotube arrays by anodization are commonly amorphous. As expected, in Figure 2, XRD patterns of samples 1 and 5 exhibit amorphous nanotube array structures. Hence, the XRD patterns of sample 4 are of considerable interest since the anatase (101) peak indicating the crystallization of the tubes. The samples 2 and 3 also exhibit slight anatase (101) peaks in the XRD patterns. At room temperature, crystallization of the tubes is influenced by some factors, such as the migration of Ti^4+^ outward from the substrate and O^2-^/OH^-^ inward to form crystalline oxide in a ratio that favors crystal growth [21], and the constraints imposed by the nanotube walls [13,22]. Sample 4 tends to form partial crystal structure since it, on one hand, prepared in the more water containing electrolyte which produced the faster ions mobility, can offer adequate ions that favor crystal growth, on the other hand, has the thinner tube walls, which impose the less constraints (in Figure 3). As to the samples 2 and 3, the slight crystallization maybe due to the internal stress relaxation of the tube wall that became thinner by dissolution after long time anodization. All XRD patterns, detected with the X-ray glancing angle at 4° show the intensive titanium diffraction peaks.

**Figure 4:**
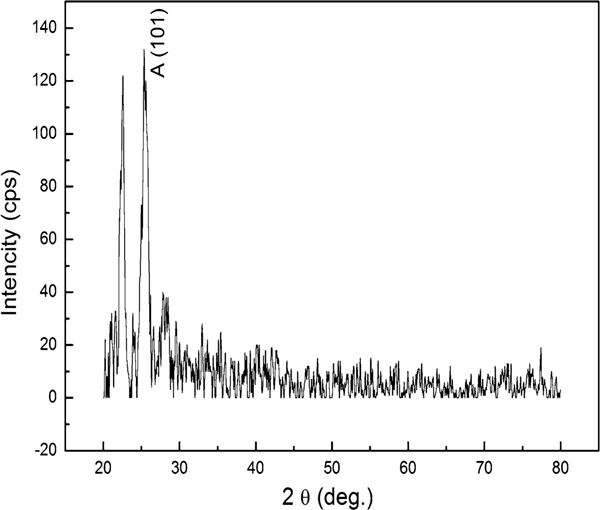
**XRD patterns of sample 4 (X-ray glancing angle 2°)**.

Que Anh S. Nguyen and co-workers reported that the barrier layer oxide is actually semi-crystalline in nature, while the tubes are amorphous in as-synthesized TiO_2_ nanotube arrays [20]. In order to get further information about the tube microstructure, XRD trace, with glancing angle of 2°, was detected for sample 4. As shown in Figure 4, due to the low sampling depth, XRD pattern that represents the surface structure information only shows the strong anatase (101) peak and another diffraction peak at two-theta of 23°. This conforms that the crystal indeed exists in the tube walls, and the diffraction peak at two-theta of 23° maybe due to the metastable anatase phase in the tubes.

### Insights into Nanotube Growth Mechanism

It has been suggested that nanotubes' growth results from the simultaneous oxidation and dissolution of the anodized oxide via F^-^ in an acidic electrolyte, described by the following reactions: [23]

(1) $$\text{Ti}+2{\text{H}}_{2}\text{O}\to {\text{TiO}}_{2}+4{\text{e}}^{-}+4{\text{H}}^{+}$$

(2) $${\text{TiO}}_{2}+6{\text{F}}^{-}+4{\text{H}}^{+}\to {\left[{\text{TiF}}_{6}\right]}^{2-}+2{\text{H}}_{2}\text{O}$$

**Figure 5:**
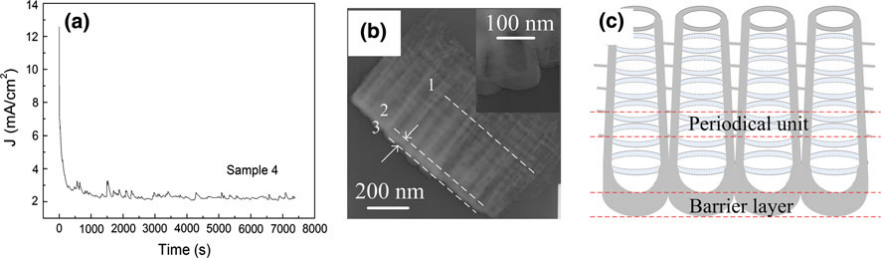
***a* Current–time curve of sample 4; *b* TEM image of sample 4 (inset showing the *bottom of a tube*), and *c* typical schematic diagram of the tube structure**.

Results from the present work indicate that the nanotube growth process could be a periodical model. Figure 5a shows the current–time curve recorded during anodization for sample 4. The current curve exhibits oscillations with asymmetric amplitude after the initial sharp slope while no steady stage as described by previous work [23,24]. Current oscillations, caused by periodical oxidation and dissolution [25], imply that the nanotube layers grow in a periodical model. But what is the periodical unit? In Figure 5b, TEM image of sample 4 shows the clear tubular structure with the ridges. From the dashed line 1, one can observe that the ridges connect and exist in a flat place perpendicular to the tube. Between dashed lines 2 and 3, it can also be found that the tube bottoms are connected to form a layer. It is reasonable to believe that the bottom layer has a close relationship with the layered ridges. The bottom thickness, in the inset of Figure 5b, is about 60 nm and almost the same size as the distance between two ridges along a tube (Figure 1d). Herein, we present a layered growth model of TiO_2_ nanotube arrays, in which the key processes can be described as follows.

(1) The first oxide layer is formed in the initial stage of anodization, and the reaction is described by Eq. 1. This rough layer endures high potential and thus some randomly distributed breakdown take place. The fast chemical dissolution of TiO_2_ at these locations results in the pits formation, as described by Eq. 2.

(2) With the increase in the pits size, the electrolyte has the chance to infiltrate into the interface of oxide layer/metal and the second oxide layer is formed and then broken down again by the electric field. Thereafter, the oxide is formed and broken down layer by layer and the current curve exhibits oscillations correspondingly. The pores are formed due to the longitudinal prolongation and the lateral expansion of the pits with the increase in oxide layer number.

(3) With the pore size increasing, the internal surface area increases and the surface tension that causes the shrinking of the pore increases, too. If the adjacent pores become close adequately, the oxide among them would be pulled apart by the surface tension, eventually leading to the pore separation and the tube formation.

(4) Because the pore size increasing in the latter layer lags that in the former layer, the pore separation in subsequent layer occurs later. Therefore, some oxide between adjacent layers would be remained and thus the ridges are formed. In Figure 5c, the schematic diagram of the tube structure illustrates the periodical unit between two ridge flats and the barrier layer formed by connected bottoms. In this case, the tube structure is the result of the barrier layer moving forward Ti substrate periodically.

## Conclusions

Highly ordered TiO_2_ nanotube arrays were anodized in electrolytes, and the morphology can be well controlled by varying anodic conditions. By mechanically bending the samples, the nanotube arrays were separated from the Ti substrate, but no obvious oxide layer has been found on the bared substrate. The as-synthesized sample, prepared in more water (Vol. 50%) containing electrolyte, exhibits anatase phase crystalline structure and is further confirmed to exist in the tube wall by low glancing angle XRD trace. It can also be found slight anatase (101) peaks in XRD traces of the samples prepared in less water (Vol. 3%) containing electrolyte but with longer anodic time. The crystal growth maybe due to the faster ions mobility, which can offer adequate ions that favor crystal growth in more water containing electrolyte or the thinner tube walls that impose the less constraints for the wall reconstruction. By analysis on current curve in conjunction with the SEM and TEM images, a layered growth model is put forth to obtain further understanding of anodic TiO_2_ nanotube arrays formation.
